# An integrative genomics approach for identifying novel functional consequences of *PBRM1* truncated mutations in clear cell renal cell carcinoma (ccRCC)

**DOI:** 10.1186/s12864-016-2906-9

**Published:** 2016-08-22

**Authors:** Yuanyuan Wang, Xingyi Guo, Michael J. Bray, Zhiyong Ding, Zhongming Zhao

**Affiliations:** 1 Department of Biomedical Informatics, Vanderbilt University School of Medicine, Nashville, TN 37203 USA; 2 Division of Epidemiology, Department of Medicine, Vanderbilt University School of Medicine, Nashville, TN 37232 USA; 3 Vanderbilt Genetics Institute, Vanderbilt University School of Medicine, Nashville, TN 37232 USA; 4 Department of Systems Biology, University of Texas MD Anderson Cancer Center, Houston, TX 77030 USA; 5 Department of Cancer Biology, Vanderbilt University School of Medicine, Nashville, TN 37232 USA; 6 Department of Psychiatry, Vanderbilt University School of Medicine, Nashville, TN 37212 USA; 7 Center for Precision Health, School of Biomedical Informatics, The University of Texas Health Science Center at Houston, Houston, TX 77030 USA

**Keywords:** Clear cell renal cell carcinoma (ccRCC), Driver gene, *PBRM1*, Expression, Methylation, microRNA

## Abstract

**Background:**

Clear cell renal cell carcinoma (ccRCC) is the most common type of kidney cancer. Recent large-scale next-generation sequencing analyses reveal that *PBRM1* is the second most frequently mutated gene harboring many truncated mutations and has a suspected tumor suppressor role in ccRCC. However, the biological consequences of *PBRM1* somatic mutations (e.g., truncated mutations) that drive tumor progression in ccRCC remain unclear.

**Methods:**

In this study, we proposed an integrative genomics approach to explore the functional consequences of *PBRM1* truncated mutations in ccRCC by incorporating somatic mutations, mRNA expression, DNA methylation, and microRNA (miRNA) expression profiles from The Cancer Genome Atlas (TCGA). We performed a systematic analysis to detect the differential molecular features in a total of 11 ccRCC samples harboring *PBRM1* truncated mutations from the 33 “pan-negative” ccRCC samples. We excluded the samples that had any of the five high-confidence driver genes (*VHL*, *BAP1*, *SETD2, PTEN* and *KDM5C*) reported in ccRCC to avoid their possible influence in our results.

**Results:**

We identified 613 differentially expressed genes (128 up-regulated and 485 down-regulated genes using cutoff |log_2_FC| < 1 and *p* < 0.05) in *PBRM1* mutated group versus “pan-negative” group. The gene function enrichment analysis revealed that down-regulated genes were significantly enriched in extracellular matrix organization (adjusted *p* = 2.05 × 10^−7^), cell adhesion (adjusted *p* = 2.85 × 10^−7^), and ion transport (adjusted *p* = 9.97 × 10^−6^). Surprisingly, 26 transcriptional factors (TFs) genes including *HOXB9, PAX6* and *FOXC1* were found to be significantly differentially expressed (23 over expressed TFs and three lower expressed TFs) in *PBRM1* mutated group compared with “pan-negative” group. In addition, we identified 1405 differentially methylated CpG sites (targeting 1308 genes, |log_2_FC| < 1, *p* < 0.01) and 185 significantly altered microRNAs (|log_2_FC| < 1, *p* < 0.05) associated with truncated *PBRM1* mutations. Our integrative analysis suggested that methylation and miRNA alterations were likely the downstream events associated with *PBRM1* truncation mutations.

**Conclusions:**

In summary, this study provided some important insights into the understanding of tumorigenesis driven by *PBRM1* truncated mutations in ccRCC. The approach may be applied to many driver genes in various cancers.

**Electronic supplementary material:**

The online version of this article (doi:10.1186/s12864-016-2906-9) contains supplementary material, which is available to authorized users.

## Background

Renal cell carcinoma (RCC) is the most common type of kidney cancer (>85 %), which causes ~3 % deaths in men in the United States every year [1, 2]. RCC can be classified into four clinical subtypes including clear cell renal cell carcinoma (ccRCC), papillary RCC (pRCC), chromophobe RCC (chRCC), and renal oncocytoma (RO). Among them, ccRCC is the most common type representing 75–85 % of all RCC cases [2, 3]. Unlike other cancer types that are found to have recurrent mutations in oncogenes [4–7], ccRCC tumors are mainly associated with somatic mutations in tumor suppressor genes such as *VHL*, *PBRM1*, *BAP1* and *SETD2* [8–10].

*PBRM1* (Polybromo-1, pb1, encoding BAF180 protein), which maps to 3p21, plays an ATP-dependent chromatin-remodeling role as a subunit of the SWI/SNF (SWItch/Sucrose Non-Fermentable) complex [11–13]. *PBRM1* is found to mediate gene regulation of cell growth, migration, proliferation and differentiation in multiple cancer types including kidney, bladder, and breast. Among these cancer types, *PBRM1* is one of the most frequently mutated and studied genes in ccRCC than any other cancer types [11, 12, 14–18]. In ccRCC, *PBRM1* is the second most frequently mutated gene; it is observed in ~40 % of tumor cases and functions as a driver tumor suppressor gene [3, 9, 10, 13, 18–20]. *PBRM1* mutations in ccRCC samples may lead to a dysregulation of several critical cell signaling pathways including actin-based motility by rho, tight junction signaling, axonal guidance signaling and germ cell-sertoli cell junction signaling [21]. Furthermore, mutations in *PBRM1* are identified as the root of tumor evolution in a subgroup of ccRCC [22]. While previous studies have focused on the exploration of particular downstream genes and pathways directly regulated by *PBRM1* gene, an in-depth integrative analysis on the biological consequences of *PBRM1* truncated mutations has not been done yet. Such an analysis is important because tumor suppressor genes play function largely through truncated mutations [23].

Here, we performed an integrative genomics analysis to investigate the biological consequences of truncated *PBRM1* mutations in ccRCC. We downloaded multiple -omics data including RNA-Seq, DNA methylation, and microRNA-Seq data of ccRCC samples from The Cancer Genome Atlas (TCGA). We systemically compared molecular features in a total of 11 mutated *PBRM1* samples with those in 33 “pan-negative” samples; and those samples were all exclusive of any of the five known ccRCC driver genes (*VHL*, *BAP1*, *SETD2*, *PTEN* and *KDM5C*) [13, 15]. The approach allowed us to maximally reduce the noise from the observed molecular signals. We identified a substantial proportion of molecular alterations including changes in gene expression, DNA methylation, and dysregulation of microRNAs (miRNAs) that were significantly associated with truncated *PBRM1* mutations, as well as the follow up pathway, co-expression network, and hypothesized mechanism analysis.

## Results

### Workflow for defining *PBRM1*-mutated and “pan-negative” sample groups

**Fig. 1:**
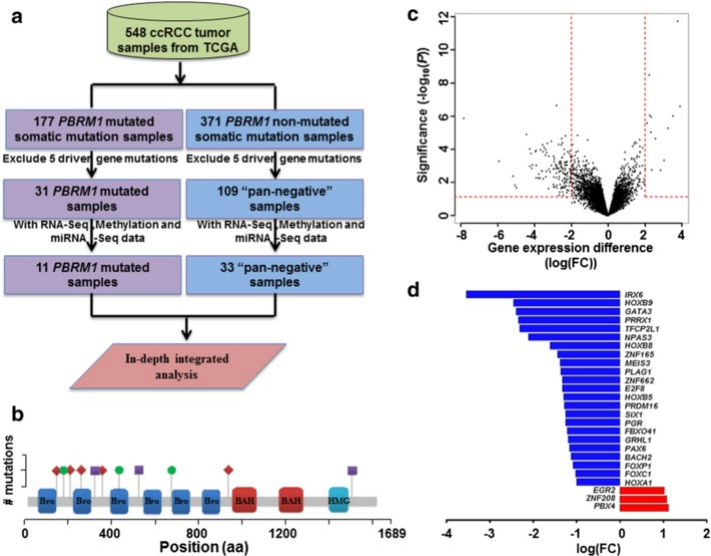
Sample filtering workflow used for integrative genomic analyses and differential expression results by comparing 11 *PBRM1* mutated and 33 “pan-negative” ccRCC samples. **a** A sample filtering workflow was used for integrative genomic analyses. First, 548 ccRCC samples were split into *PBRM1* mutated group (177 samples) and *PBRM1* non-mutated group (371 samples). Five high-confidence ccRCC driver genes (*VHL*, *BAP1*, *SETD2*, *PTEN* and *KDM5C*) were excluded in both groups, resulting in 31 *PBRM1* mutated samples and 109 “pan-negative” samples. After that, samples that have all DNA methylation, RNA-Seq, and miRNA-Seq data were extracted; resulting in 11 *PBRM1* mutated samples and 33 “pan-negative” samples for further in-depth integrative analysis. **b** Cartoon representation of mutation types and locations in 11 *PBRM1* truncation mutated samples. Five nonsense mutations (red diamond), three splice sites mutations (green round), and three frame shift deletions (purple square) were observed in 11 *PBRM1* truncated mutation samples. **c** Volcano plot of significance of gene expression difference between *PBRM1* mutated group and “pan-negative” group at gene expression levels. Each dot represents one gene. The x axis shows the gene expression difference by a log transformed fold change while the y axis shows significance by –log_10_ transformed *p*-value value obtained from edgeR. A gene is called significantly and differentially expressed if its |log(FC)| > 2 and *p*-value < 0.05. Red dashed line shows |log(FC)| =2 or *p*-value = 0.05. **d** Bar plot of log transfer of fold change in differentially expressed transcriptional factors. 23 transcriptional factors were found to be down-regulated in *PBRM1* mutated group while three transcriptional factors were found up-regulated

Somatic mutation profiles for 548 tumor samples in ccRCC, or kidney renal clear cell carcinoma (KIRC), were downloaded from TCGA (data accessed on January 20, 2015). After examining *PBRM1* mutations, we separated samples into two groups including 177 mutated *PBRM1* samples and 371 non-mutated *PBRM1* samples, respectively (Additional file 1) [13]. We further excluded a total of 146 and 262 samples for downstream analysis because they carried mutations in five high-confidence ccRCC driver genes (*VHL, BAP1, SETD2, PTEN,* and *KDM5C*) [13, 15]. This process resulted in 31 *PBRM1* mutated samples and 109 “pan-negative” samples, respectively (Fig. 1a, Additional file 1). In the next step, we identified the samples with matched RNA-Seq, DNA methylation, and microRNA-Seq data; this resulted in a total of 11 mutated *PBRM1* samples and 33 “pan-negative” samples. They were used for the analyses for downstream pre-transcriptional and transcriptional events (Fig. 1a, and b, Additional file 1). Importantly, those 11 samples carried “loss of function” mutations in *PBRM1* gene, including five nonsense mutations, three splice sites mutations and three frame shift deletions (Fig. 1b, Additional file 2: Table S1).

### Identification of transcriptional factors from differentially expressed genes associated with *PBRM1* truncated mutations

**Fig. 2:**
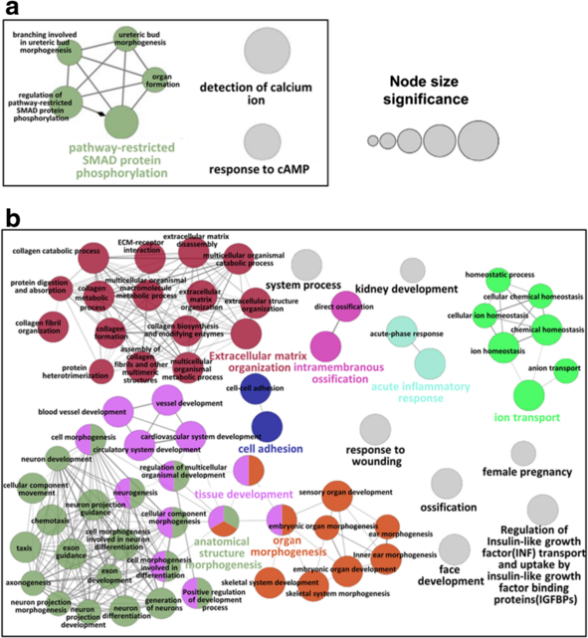
Functional enrichment results of differentially expressed genes from RNA-Seq of *PBRM1* mutation ccRCC samples. **a** Clustered function and pathway enrichment results of up-regulated genes in *PBRM1* mutated group compared with “pan-negative” group, with *p*-value < 0.01 results shown. Different clusters were shown in different colors. **b** Clustered function and pathway enrichment results of down-regulated genes in *PBRM1* mutated group compared with “pan-negative” group, with *p*-value < 0.001 results shown. Different clusters were shown in different colors

We performed a comparative analysis on gene expression profiles to identify the differential expressed genes (DEGs) between the *PBRM1* mutated group and “pan-negative” group using edgeR [24]. At a significance threshold of absolute log_2_ transferred fold change (|log_2_FC|) > 1 and *p* < 0.05, a total of 613 DEGs were identified including 128 genes having over expression and 485 genes showing lower expression in *PBRM1* mutated samples compared with the “pan-negative” group (Fig. 1c, Additional file 1 and Additional file 3). Of those DEGs, 26 transcription factors were observed, 23 were down-regulated but only three were up-regulated (Fig. 1d). Interestingly, four Antp homeobox family and two forkhead family transcriptional factors (HOXA1, HOXB5, HOXB8, HOXB9, FOXP1, and FOXC1) that are involved in cell development and proliferation [25] were found to be down-regulated in the *PBRM1* mutated group versus “pan-negative” group. Additionally, GATA3, a transcription factor that was observed to be down-regulated in *PBRM1* mutated group in our study, was previously found to be an important early event and potential regulator that associated with loss of TGFβ receptor expression in ccRCC [26, 27] (Fig. 1d). Gene function enrichment analysis showed that down-regulated genes were significantly enriched in extracellular matrix organization (adjust *p* = 2.05 × 10^−7^), cell adhesion (adjust *p* = 2.82 × 10^−7^) and ion transport (adjust *p* = 1.61 × 10^−5^), while up-regulated genes were significantly enriched in pathway-restricted SMAD protein phosphorylation (adjust *p* = 3.59 × 10^−3^) (Fig. 2a and b, Additional file 2: Tables S2 and S3, Additional file 3). We further examined gene expression and methylation, as hypo-methylation is often related to active transcription and gene expression. Our examination the relationship between lower expressed genes and hyper-methylated genes showed that 33 down-regulated genes were hyper-methylated (we abbreviated as hyper-down genes), including BCAT1 associated with cell growth, HOXB9 encoding a cell cycle regulation transcription factor, and *PAX6* encoding a cellular development associated transcription factor (Additional file 1) [25].

### Widespread epigenetic silencing associated with *PBRM1* truncated mutations

**Fig. 3:**
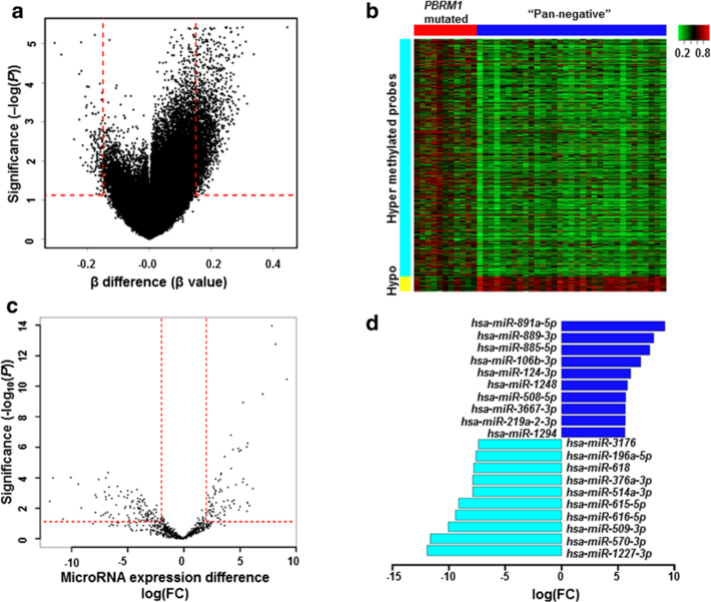
Methylation pattern and miRNA expression pattern in *PBRM1* mutated ccRCC **a** Volcano plot of significance of DNA methylation pattern difference (β-difference) between *PBRM1* mutated group and “pan-negative” group. Each dot represents one methylation probes. The x axis shows the difference in β-value (β-difference) while the y axis shows the significance by –log transformed *p-*value obtained from Samr. A probe is called significantly and differentially expressed if its |β-difference| > 0.15 and *p*-value < 0.01. The red dashed line shows |β-difference| =0.15 or *p*-value = 0.01. **b** Heat map of differential expressed methylation probes between *PBRM1* mutated group and “pan-negative” group. **c** Volcano plot of significance of miRNA expression differences between *PBRM1* mutated group and “pan-negative” group. Each dot represents one miRNA. The x axis shows log transformed fold changes of miRNA expression while the y axis shows significance by –log_10_ transformed *p*-value obtained from edgeR. A probe is called significantly and differentially expressed if its |log(FC)| > 1 and *p*-value < 0.05. Red dashed line |log(FC)| =1 or *p*-value = 0.05. **d** Bar plot of top ten up-regulated miRNAs and down-regulated miRNAs that revealed in *PBRM1* mutated samples compared with “pan-negative” ccRCC samples

We analyzed genome-scale DNA methylation profiles by comparing β-value changes (measured as β-differences) between mutated *PBRM1* group and “pan-negative” group (see Methods). A total of 1308 differentially methylated genes covering 1405 differentially methylated CpG sites were identified using |β-difference| > 0.15 and *p* < 0.05 cutoff (Fig. 3a and b, Additional file 1, Additional file 4: Figure S1). Among those genes, 1229 hyper-methylated (94 %) and 79 hypo-methylated genes (6 %) were observed in *PBRM1* mutated samples compared to the “pan-negative” samples, suggesting that an global gene inactivation may be associated with *PBRM1* truncated mutations (Additional file 2: Table S4, Additional file 4: Figure S2). This observation is consistent with the differential gene expression results above (more down-regulated genes than up-regulated genes in *PBRM1* group); however, these genes may not be immediately regulated by *PBRM1* because truncated mutations in a tumor suppressor gene are expected to result in up-regulation of its immediately regulated gene according to the “loss of function” model. Functional enrichment analyses indicated that those hyper-methylated genes were significantly enriched in multiple processes including generation of neurons (q = 1.20 × 10^−5^), cell differentiation (q = 1.22 × 10^−5^), and regulation of catabolic process (q = 4.02 × 10^−5^), while glomerulus development was observed to be most significant in hypo-methylated genes (q = 3.21 × 10^−3^) (Additional file 2: Tables S5 and S6, Additional file 4: Figure S2). Interestingly, we found that hyper-methylated CpG sites exhibited a significantly higher proportion residing in several gene regions including promoters and gene bodies than hypo-methylated genes (Additional file 4: Figure S3) [28].

### miRNA dysregulation associated with *PBRM1* truncation mutations

**Fig. 4:**
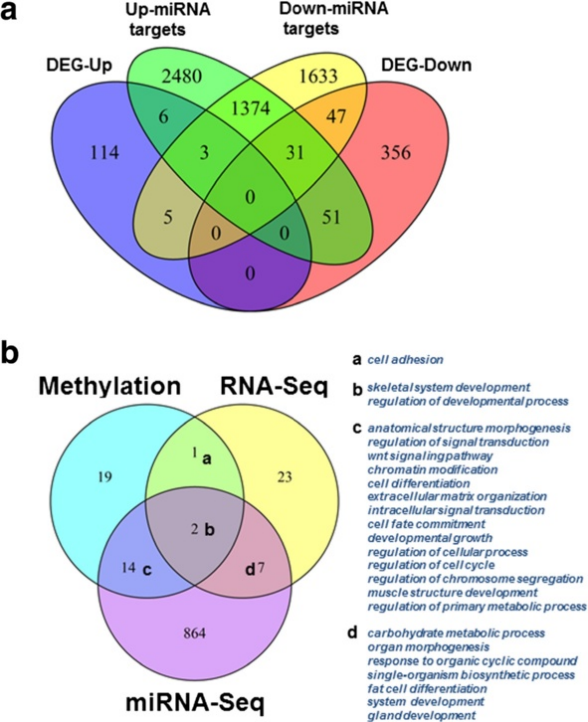
Integrative analysis results of function terms and pathway enrichment. **a** Venn representation of the overlaps among up-regulated genes (DEG-up), down-regulated genes (DEG-down), target genes of up-regulated miRNAs (Up miRNA targets) and target genes of down-regulated miRNAs (Down miRNA targets). **b** Venn representation of overlaps among function and pathway enrichment results from differential methylated genes (methylation), differential expressed genes (RNA-Seq) and targets genes of differential expressed miRNA (miRNA-Seq)

A total of 185 differentially expressed miRNAs were identified to be associated with *PBRM1* truncation mutations using the cutoffs: absolute log_2_ transferred fold change (|log_2_FC|) > 1 and *p* < 0.05. Among them, 87 miRNAs exhibited up-regulation pattern in *PBRM1* mutated samples while the remaining 98 miRNAs exhibited down-regulation pattern (Fig. 3c, Additional file 1). The 10 most differentially expressed miRNAs were shown in Fig. 3d. Interestingly, three identified miRNAs (miR-221, miR-222 and miR-16) exhibiting down-regulation patterns in *PBRM1* mutated group were consistent with the previous reports by experimental studies [13]. Next, we performed the analysis of those predicted targets genes that may be regulated by these differentially expressed miRNAs. Among the differentially expressed miRNAs, 64 up-regulated miRNAs and 56 down-regulated miRNAs had targets in TarBase [29] or miRTarBase [30] database. We observed 3093 and 3945 target genes for up-regulated miRNAs and down-regulated miRNAs, respectively. Comparisons between miRNA targets and DEGs revealed that 14 miRNA target genes were up-regulated while 129 were down-regulated, in which nine miRNA target genes were hyper-methylated and also down-regulated in *PBRM1* mutated group (Fig. 4a, Additional file 1). Functional enrichment analysis revealed that 24 functional terms and pathways, including extracellular matrix organization and extracellular structure organization pathways, were observed in more than one gene set; and these gene sets are differentially expressed genes, differentially methylated genes, and differential expressed miRNA targets genes (Fig. 4b).

### Integrated analysis for *PBRM1* truncated mutations in ccRCC

**Fig. 5:**
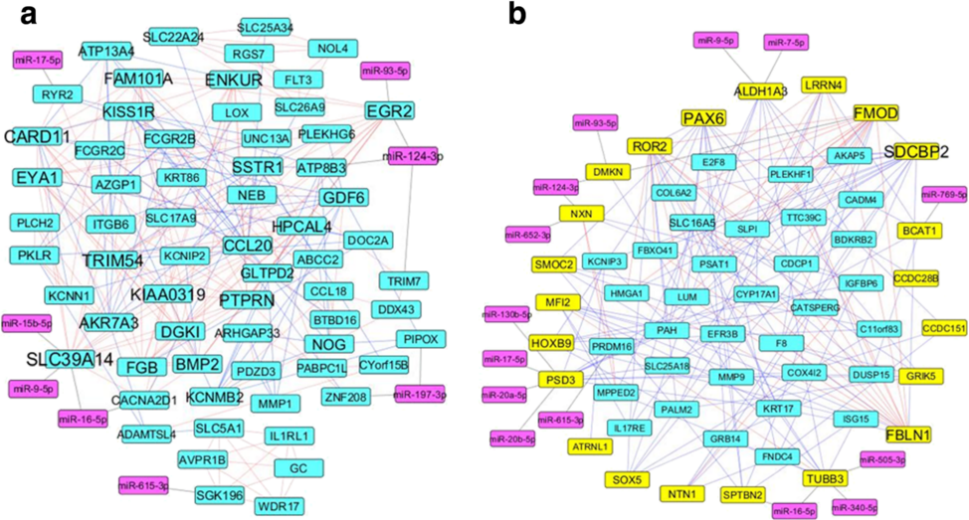
*PBRM1* mutation specific, up-regulated and down-regulated co-expression network. Highly co-expressed genes in *PBRM1* mutated groups were mapped into a protein-protein interaction network from PINA2, as reference network. 128 up-regulated genes and 33 hyper-down (hyper-methylated and down-regulated) genes were mapped into the reference network, as up-regulated co-expression network (**a**) and down-regulated core co-expression network. **b** In down-regulated core co-expression network, only first neighbors of 33 hyper-down genes in down-regulated genes were kept in network. In both networks, only genes with degree above there were kept for better version

To further explore the regulatory mechanisms of the identified genes and miRNAs associated with *PBRM1* truncated mutations in ccRCC, we constructed co-expression networks using R software based on mRNA expression results (Fig. 5a and b, detailed information is in *Methods*). To identify miRNAs that involved in gene co-expression networks, miRNAs target genes that were found co-expressed with other genes and corresponding miRNAs were also included in co-expression networks. Six miRNAs (*miR-17-5p, miR-9-5p, miR-16-5p, miR-615-3p, miR-124-3p,* and *miR-93-5p*) were observed in both up-regulated and down-regulated co-expression networks, in which different possible targets were involved. The miRNA target genes including *SLC39A14* and *EGR2* that are related to ion transport and cell growth were observed in the *PBRM1*-specific up-regulated co-expression network, suggesting that miRNAs may be involved in ion transport and a cell growth process in *PBRM1*-driven dysregulation. In the *PBRM1* specific down-regulated co-expression network, two down-regulated DNA-binding transcription factors HOXB9 and PAX6 were observed as positively co-expressed with several genes and regulated by miRNAs, suggesting their essential role in *PBRM1*-related down-regulation (Additional file 1). Similarly, *SDCBP2* and *PAX6* were found to be positively co-expressed with many genes in the down-regulated co-expression network (Additional file 1), which further verified the association of compound metabolisms and development with *PBRM1* truncation mutations [25].

**Fig. 6:**
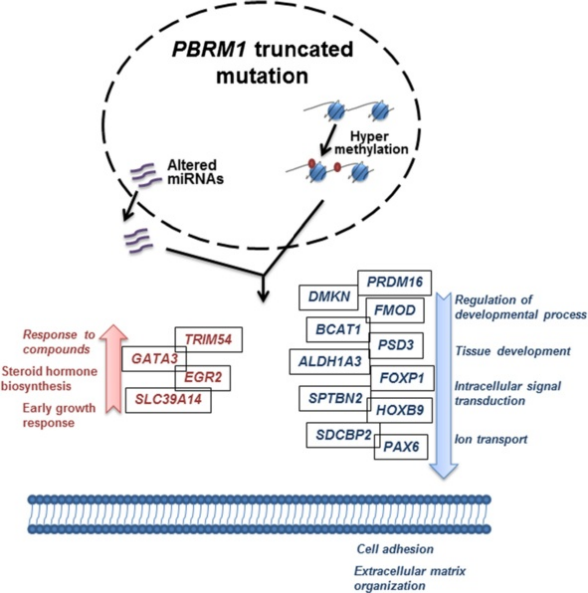
Hypothesized mechanisms of *PBRM1* truncated mutation functions in the tumor genetics of ccRCC. Hyper-methylation and altered miRNAs expression were found associated with *PBRM1* truncated mutation in ccRCC. Up-regulated genes and pathways were shown in red (*left*) while down-regulated genes and pathways were shown in blue (*right*)

Collectively, *PBRM1* truncated mutations may lead to the pre-transcriptional deregulation at DNA methylation level and the post-transcriptional deregulation at the miRNA expression level. Accordingly, this resulted in widespread hyper-methylation and miRNA expression alteration in ccRCC tumor genomes (Fig. 5). Based on our integrative genomic analysis results, we proposed the possible regulations linked to the *PBRM1* truncated mutations in the tumorigenesis of ccRCC (Fig. 6). These functional alterations include both up-regulation and down-regulation of molecules and pathways that are associated with the miRNA and methylation changes in *PBRM1*-truncated mutation tumor cells.

## Discussion

This study highlights the association between *PBRM1* truncated mutations and decreased extracellular matrix organization, cell adhesion, ion transport and tissue development. This suggests that *PBRM1* plays an important regulatory role in cell-cell crosstalk in the tumorigenesis of ccRCC. In this study, there are more differentially methylated genes (1308 genes) than differential expressed genes (613 genes) in *PBRM1* mutated group, suggesting a complicated pre-transcriptional level regulation with DNA methylation involved in *PBRM1* mutations.

Studying the downstream events of a driver gene has become important now because the scientific community has witnessed large amount of genomic data allowing the sample stratification by driver mutation and also because a driver gene may lead to many critical biological events linking to tumorigenesis or drug treatment [31, 32]. We recently develop approaches to study the downstream events of a specific mutation in a driver gene (BRAF^V600E^ and NRAS^Q61^) in melanoma [4, 5]. To our knowledge, this is the first study to integrate pre-transcriptional and post-transcriptional level data to investigate the main effects of a driver gene (*PBRM1*) through its truncated mutations in a cancer (ccRCC). Observations in this study are based on 11 *PBRM1* mutated and 33 “pan-negative” ccRCC samples, which may have some bias because of the small sample size. However, by an integrative analysis of multiple -omics data, we could still achieve reliable results for further validation. As we did similarly in melanoma [4, 5], the stratification of samples by driver mutation only (cases) and “pan-negative” samples (controls) would likely increase the power because it effectively removed the noise from similar samples with other driver mutations. This is especially important in cancer genomics studies because driver mutations may affect the same or similar signaling pathways (e.g., Ras pathway). Our results suggest that *PBRM1* mutations are an important event in the early stage of ccRCC tumor genetics, which paves the way for further *PBRM1*-related research in ccRCC. To excluded the influences of other driver genes and highlight the effects of *PBRM1* in ccRCC, we defined the “pan-negative” ccRCC sample set by excluding samples that contained somatic mutations in any of the five well-known driver genes in ccRCC. Future validation may apply the similar strategies. Our integrative analysis using methylation, gene expression, and miRNA expression is the first to study the *PBRM1* truncation mutation specific dysfunction in co-expressed networks. All mutations in 11 *PBRM1* mutated samples are truncation mutations, which signify dysfunction state of *PBRM1* as a tumor suppressor gene in ccRCC.

There are several limitations in this study. First, how our results are related to the influence of *PBRM1* on tumor prognosis needs further investigation because previous studies suggest the association between *PBRM1* mutations and prognosis of ccRCC is still unclear [13, 22, 33, 34]. In addition, copy number variants of *PBRM1* are not considered either since we only focus on the downstream consequences that associate with early somatic mutation events in *PBRM1*. No validation cohorts of *PBRM1* have involved in this study yet because of the limited results available related to *PBRM1* at the current stage. We hope more reports will become available from other groups in the near future so that our results may be experimentally validated. Our analysis focuses on the gene level changes that associated with *PBRM1* truncated mutation, in which protein level changes were not considered because of the complicated regulation from gene expression level to protein level.

*PBRM1* is found to be highly mutated in several cancer types. It is most frequently mutated in ccRCC. Loss of function and expression of *PBRM1* was less common in non-ccRCC than in ccRCC, suggesting a specific regulatory role of *PBRM1* truncation mutations in ccRCC [35]. In breast cancer, *PBRM1* is shown to be a core regulator of p21 [14]; however, we could not find a similar pattern in ccRCC. The result suggests that *PBRM1* may act differently through its regulation mechanisms in different cancer types. Future studies to dissect the role of *PBRM1* in different cancer types would be helpful to better understand the mechanisms of *PBRM1* truncation mutations and tumorigenesis. More cancer genomic data is expected from large consortia like the International Cancer Genome Consortium (ICGC). So, a follow up study is needed in future.

## Conclusion

Our study investigated molecular alterations including gene expression, methylation, and miRNA expression that associated with *PBRM1* truncation mutations in clear cell renal cell carcinoma. Our analysis results identified 613 differentially expressed genes, 1308 differentially methylated genes and 185 differentially expressed miRNAs between *PBRM1* mutated group and “pan-negative” group. Hypothesized mechanisms of *PBRM1* mutations in ccRCC were explored based on the integrative analysis results. Our results provide some important insights into the *PBRM1* regulation in the tumor development of ccRCC.

## Methods

### Summary of ccRCC samples

A total of 548 ccRCC (KIRC) samples were downloaded from TCGA. Level 2 results from both BI Mutation Calling and BCM Mutation Calling were utilized to find somatic mutations in all samples. 177 of 548 ccRCC samples (32.3 %) were identified to have *PBRM1* mutations and 371 samples (67.7 %) were identified as *PBRM1* non-mutated or control samples. To eliminate the influence of other driver genes, five well-known mutation genes (*VHL, BAP1, SETD2, PTEN and KDM5C*) were suggested as highly potential driver genes of ccRCC based on the somatic mutation results and earlier researches [13]. Samples with somatic mutations of those five genes were excluded from both mutated and non-mutated *PBRM1* samples, resulting in 31 *PBRM1* mutated samples and 109 “pan-negative” samples (Fig. 1a). Finally, 11 *PBRM1* mutated samples and 33 “pan-negative” samples that had DNA methylation, gene expression, and miRNA expression data were utilized for all the analyses in this study.

### RNA-Seq and miRNA-Seq data pre-processing and differential expression analysis

RNA-Seq and miRNA-Seq data were downloaded from IlluminaHiSeq_RNASeqV2 and BCGSC IlluminaHiSeq_miRNASeq platform in TCGA database, respectively. Level 3 data were utilized to find RNA expression and miRNA expression. In each group, genes/miRNAs with no expression were removed, while only genes/miRNAs with counts per million (cpm) >1 in at least two samples were kept for further analysis. edgeR package [24] in R software was used in differential RNA-Seq and miRNA expression analysis. We defined significantly DEGs or differentially expressed miRNAs if they had |log_2_FC| > 1 and *p* < 0.05. MiRNA target genes were retrieved from databases TarBase [29] and miRTarBase [30].

### Methylation analysis

Illumina HumanMethylation450K BeadChip Kit containing 486,428 CpG sites was used to explore DNA methylation profile on the genome scale. Probes targeting the X and Y chromosome, probes containing a single-nucleotide polymorphism (SNP) within five base pairs of CpG site, and probes that had no reference gene location were also removed. In total, 312,777 probes were kept for further analysis. β-values that ranged between 0 and 1 were used to represent the relative methylation level, which was measured as logistic transformation of the ratio of the methylated probe intensity over all methylation probe intensities [36]. β-difference value (differences between β-values) was used to characterize different methylation levels between *PBRM1* mutated group and non-mutated “pan-negative” group. All methylation analysis was performed in R/Bioconductor packages [37]. Samr package in R software [37] was used to calculate the significance of each CpG site. Probes with |β-difference| > 0.15 and *p* < 0.01 were selected as differentially methylated probes, and the gplots package in R software was used to obtain a heatmap of differentially methylated probes.

### Gene function and pathway enrichment analysis

The ClueGO plugin [38] in Cytoscape software [39] was used for gene function and pathway enrichment analysis. Catalogues in GO Biological Process, KEGG, REACTOME and WikiPathways databases that catalogued in ClueGo were applied for the functional enrichment analysis. The Benjamin-Hochberg method [40] was used in the adjustment of *p* (false discovery rate), and other parameters were retained as default in GlueGO. Gene sets or pathways with adjusted *p* < 0.05 were retained for further analysis. Transcription factors were annotated based on the TRANSFAC database (downloaded on April 1, 2015) [41].

### *PBRM1* mutation specific, differentially regulated co-expression network

The Pearson correlation coefficient in R software was used to calculate the correlation of each pair on all the 14270 genes that were extracted from RNA-Seq results after excluding low expression genes in *PBRM1* mutated group. The top 5 % co-expressed gene pairs were kept as co-expressed and protein-protein interactions from PINA2 [42] were used to find out the relationships between co-expressed genes, which resulted in a *PBRM1* mutation specific background network that contains 335,726 gene interaction pairs. 128 up-regulated genes and miRNAs with targets in up-regulated genes were mapped into the reference network, resulting in a *PBRM1* mutation specific, up-regulated co-expression network. 485 down-regulated genes and miRNAs with targets in down-regulated genes were mapped into the reference network, resulting in a *PBRM1* mutation specific, down-regulated co-expression network. To explore the essential genes associated with *PBRM1* mutations, only 33 hyper-down genes and their first neighbors were kept, resulting in *PBRM1* mutation specific, down-regulated core co-expression network. The Cytoscape software was used to make the network visualization, with genes that have three or more degrees being shown in Fig. 5a and b.

## Abbreviations

ccRCC, clear-cell renal cell carcinoma; KIRC, kidney renal clear cell carcinoma; TCGA, the cancer genome Atlas.

## Additional files

Additional file 1: Related sample IDs, differential methylated gene IDs, differential expressed gene IDs and altered miRNAs and their target gene IDs. (XLS 493 kb) Additional file 2: Table S1. Detailed information of somatic mutations in 11 *PBRM1* mutated ccRCC samples. **Table S2.** Functional and pathway enrichment results of up-regulated genes. **Table S3.** Functional and pathway enrichment results of down-regulated genes. **Table S4.** Alterations of β-value distributions in *PBRM1* mutated group and “pan-negative” group. **Table S5.** Top 20 GO Terms in functional enrichment results of hyper-methylated genes. **Table S6.** Functional enrichment results of hypo-methylated genes. (DOCX 31 kb) Additional file 3: Detailed information and functional enrichment results of 128 up-regulated genes and 485 down-regulated genes. (XLS 399 kb) Additional file 4: Figure S1. Global methylation density in *PBRM1* mutated group and “pan-negative” group. **Figure S2.** Statics results of altered methylated genes numbers and functions. **Figure S3.** Percentage of different methylated CpG island region (promoter, 5’UTR, first exon, gene body and 3’UTR) in hyper-methylated and hypo-methylated genes. (DOCX 1378 kb)
