# Erratum to: An integrative genomics approach for identifying novel functional consequences of *PBRM1* truncated mutations in clear cell renal cell carcinoma (ccRCC)

**DOI:** 10.1186/s12864-016-3141-0

**Published:** 2016-10-21

**Authors:** Yuanyuan Wang, Xingyi Guo, Michael J. Bray, Zhiyong Ding, Zhongming Zhao

**Affiliations:** 1 Department of Biomedical Informatics, Vanderbilt University School of Medicine, Nashville, TN 37203 USA; 2 Division of Epidemiology, Department of Medicine, Vanderbilt University School of Medicine, Nashville, TN 37232 USA; 3 Vanderbilt Genetics Institute, Vanderbilt University School of Medicine, Nashville, TN 37232 USA; 4 Department of Systems Biology, University of Texas MD Anderson Cancer Center, Houston, TX 77030 USA; 5 Department of Cancer Biology, Vanderbilt University School of Medicine, Nashville, TN 37232 USA; 6 Department of Psychiatry, Vanderbilt University School of Medicine, Nashville, TN 37212 USA; 7 Center for Precision Health, School of Biomedical Informatics, The University of Texas Health Science Center at Houston, Houston, TX 77030 USA

## Erratum

Following publication of the original article [1] it was brought to our attention that there were three errors in the “Results” section of the article’s abstract. Please see below for the two sentences in which these errors occur:

1. We identified 613 differentially expressed genes (128 up-regulated and 485 down-regulated genes using cutoff |log_2_FC| < 1 and *p* < 0.05) in *PBRM1* mutated group versus “pan-negative” group.

2. In addition, we identified 1405 differentially methylated CpG sites (targeting 1308 genes, |log_2_FC| < 1, *p* < 0.01) and 185 significantly altered microRNAs (|log_2_FC| < 1, *p* < 0.05) associated with truncated *PBRM1* mutations.

In both sentences the present expression in the article is "|log_2_FC| < 1". However, this is incorrect, as the cutoff should be "|log_2_FC| > 1".

## References

[1] Wang Y (2016). An integrative genomics approach for identifying novel functional consequences of *PBRM1* truncated mutations in clear cell renal cell carcinoma (ccRCC). BMC Genomics.

