# Supplementary material for: An integrative genomics approach for identifying novel functional consequences of PBRM1 truncated mutations in clear cell renal cell carcinoma (ccRCC)
Source: BMC Genomics. 2016 Aug 22;17(Suppl 7):515. doi: 10.1186/s12864-016-2906-9 (PMC5001239; doi:10.1186/s12864-016-2906-9)
Supplement: Additional file 2: Table S1. — Detailed information of somatic mutations in 11 PBRM1 mutated ccRCC samples. Table S2. Functional and pathway enrichment results of up-regulated genes. Table S3. Functional and pathway enrichment results of down-regulated genes. Table S4. Alterations of β-value distributions in PBRM1 mutated group and “pan-negative” group. Table S5. Top 20 GO Terms in functional enrichment results of hyper-methylated genes. Table S6. Functional enrichment results of hypo-methylated genes. (DOCX 31 kb) [file 12864_2016_2906_MOESM2_ESM.docx]

**Table S1 Detailed information of somatic mutations in eleven ccRCC samples harboring *PBRM1* truncated mutations**

| **Source** | **Chr.** | **Start_Position** | **End_Position** | **Strand** | **Variant_Classification** | **Variant_Type** | **Reference_Allele** | **Tumor_Seq_Allele1** | **Tumor_Seq_Allele2** | **Tumor_Sample_Barcode** |
| --- | --- | --- | --- | --- | --- | --- | --- | --- | --- | --- |
| broad.mit.edu | 3 | 52651547 | 52651548 | + | Frame_Shift_Del | DEL | TC | TC | - | TCGA-B0-5712-01A-11D-1669-08 |
| broad.mit.edu | 3 | 52692214 | 52692214 | + | Splice_Site | DEL | C | C | - | TCGA-B4-5844-01A-11D-1669-08 |
| broad.mit.edu;hgsc.bcm.edu;ucsc.edu | 3 | 52588818 | 52588818 | + | Frame_Shift_Del | DEL | G | G | - | TCGA-CZ-5456-01A-01D-1501-10 |
| broad.mit.edu;hgsc.bcm.edu | 3 | 52663052 | 52663052 | + | Splice_Site | SNP | C | C | A | TCGA-CW-5589-01A-01D-1534-10 |
| broad.mit.edu;hgsc.bcm.edu;ucsc.edu | 3 | 52643561 | 52643561 | + | Nonsense_Mutation | SNP | G | G | A | TCGA-A3-3387-01A-01D-1534-10 |
| broad.mit.edu;hgsc.bcm.edu;ucsc.edu | 3 | 52668807 | 52668807 | + | Nonsense_Mutation | SNP | G | G | C | TCGA-B0-4815-01A-01D-1501-10 |
| broad.mit.edu;hgsc.bcm.edu;ucsc.edu | 3 | 52677265 | 52677265 | + | Frame_Shift_Del | DEL | G | G | - | TCGA-CZ-5453-01A-01D-1501-10 |
| broad.mit.edu;ucsc.edu | 3 | 52692235 | 52692235 | + | Nonsense_Mutation | SNP | G | A | A | TCGA-B0-5694-01A-11D-1534-10 |
| hgsc.bcm.edu;ucsc.edu | 3 | 52692298 | 52692298 | + | Nonsense_Mutation | SNP | G | G | A | TCGA-DV-5566-01A-01D-1534-10 |
| broad.mit.edu;hgsc.bcm.edu | 3 | 52692333 | 52692333 | + | Splice_Site | SNP | T | T | A | TCGA-B4-5377-01A-01D-1501-10 |
| broad.mit.edu;hgsc.bcm.edu;ucsc.edu | 3 | 52696199 | 52696199 | + | Nonsense_Mutation | SNP | C | C | A | TCGA-CZ-5454-01A-01D-1501-10 |

**Table S2 Functional and pathway enrichment results of up-regulated genes**

| **GO ID** | **GO term** | **Term *p*-value corrected by Benjamini-Hochberg** |
| --- | --- | --- |
| GO:0005513 | detection of calcium ion | 5.06E-04 |
| GO:0051591 | response to cAMP | 3.41E-03 |
| GO:0060389 | pathway-restricted SMAD protein phosphorylation | 3.59E-03 |
| REACTOME:150366 | Elastic fibre formation | 4.46E-03 |
| KEGG:00140 | Steroid hormone biosynthesis | 5.75E-03 |
| WP:24 | Peptide GPCRs | 6.56E-03 |
| KEGG:05412 | Arrhythmogenic right ventricular cardiomyopathy (ARVC) | 7.17E-03 |

**Table S3 Functional and pathway enrichment results of down-regulated genes**

| **GO ID** | **GO term** | **Term *p*-value corrected by Benjamini-Hochberg** |
| --- | --- | --- |
| REACTOME:118779 | Extracellular matrix organization | 2.05E-07 |
| GO:0007155 | cell adhesion | 2.82E-07 |
| GO:0009653 | anatomical structure morphogenesis | 9.45E-07 |
| GO:0009887 | organ morphogenesis | 1.70E-06 |
| GO:0006811 | ion transport | 9.97E-06 |
| GO:0003008 | system process | 1.04E-05 |
| GO:0009888 | tissue development | 1.61E-05 |
| GO:0001957 | intramembranous ossification | 1.11E-04 |
| GO:0002526 | acute inflammatory response | 1.16E-04 |
| REACTOME:15428 | Regulation of Insulin-like Growth Factor (IGF) Transport and Uptake by Insulin-like Growth Factor Binding Proteins (IGFBPs) | 1.34E-04 |
| GO:0009611 | response to wounding | 2.28E-04 |
| GO:0001503 | ossification | 4.30E-04 |
| GO:0060324 | face development | 4.58E-04 |
| GO:0007565 | female pregnancy | 6.13E-04 |
| GO:0001822 | kidney development | 6.58E-04 |

**Table S4 Alterations of β values distribution in PBRM1 mutated group and “pan-negative” group**

| **β value** | ***PBRM1* mutated group (%)** | **“Pan-negative” group (%)** |
| --- | --- | --- |
| <0.2 | 42.0 | 43.3 |
| 0.2-0.4 | 8.05 | 7.22 |
| 0.4-0.6 | 7.87 | 8.46 |
| 0.6-0.8 | 12.0 | 13.3 |
| >0.8 | 30.1 | 27.8 |

**Table S5 Functional enrichment results of hyper-methylated genes**

| **GO ID** | **GO term** | **Term *p*-value corrected by Benjamini-Hochberg** |
| --- | --- | --- |
| GO:0048699 | generation of neurons | 1.20E-05 |
| GO:0030154 | cell differentiation | 1.22E-05 |
| GO:0009894 | regulation of catabolic process | 4.02E-05 |
| GO:0035556 | intracellular signal transduction | 6.77E-05 |
| GO:0071363 | cellular response to growth factor stimulus | 7.17E-05 |
| GO:0006928 | cellular component movement | 7.20E-05 |
| GO:0010646 | regulation of cell communication | 1.44E-04 |
| GO:0051128 | regulation of cellular component organization | 1.79E-04 |
| GO:0009653 | anatomical structure morphogenesis | 3.47E-04 |
| GO:0048864 | stem cell development | 6.38E-04 |
| GO:0022603 | regulation of anatomical structure morphogenesis | 7.47E-04 |
| GO:0070102 | interleukin-6-mediated signaling pathway | 8.91E-04 |
| GO:0031146 | SCF-dependent proteasomal ubiquitin-dependent protein catabolic process | 2.00E-03 |
| GO:1901575 | organic substance catabolic process | 3.30E-03 |
| GO:0016055 | wnt signaling pathway | 3.67E-03 |
| GO:0007268 | synaptic transmission | 4.47E-03 |
| GO:0030198 | extracellular matrix organization | 4.47E-03 |
| GO:0045165 | cell fate commitment | 4.57E-03 |
| GO:0044267 | cellular protein metabolic process | 4.89E-03 |
| GO:1901379 | regulation of potassium ion transmembrane transport | 5.69E-03 |

**Table S6 Functional enrichment results of hypo-methylated genes**

| **GO ID** | **GO term** | **Term *p*-value corrected by Benjamini-Hochberg** |
| --- | --- | --- |
| GO:0042475 | odontogenesis of dentin-containing tooth | 1.99E-03 |
| GO:0032835 | glomerulus development | 3.21E-03 |
| GO:0050810 | regulation of steroid biosynthetic process | 3.77E-03 |
