# Supplementary material for: An integrative genomics approach for identifying novel functional consequences of PBRM1 truncated mutations in clear cell renal cell carcinoma (ccRCC)
Source: BMC Genomics. 2016 Aug 22;17(Suppl 7):515. doi: 10.1186/s12864-016-2906-9 (PMC5001239; doi:10.1186/s12864-016-2906-9)
Supplement: Additional file 4: Figure S1. — Global methylation density in PBRM1 mutated group and “pan-negative” group. Figure S2. Statics results of altered methylated genes numbers and functions. Figure S3. Percentage of different methylated CpG island region (promoter, 5’UTR, first exon, gene body and 3’UTR) in hyper-methylated and hypo-methylated genes. (DOCX 1378 kb) [file 12864_2016_2906_MOESM4_ESM.docx]

**
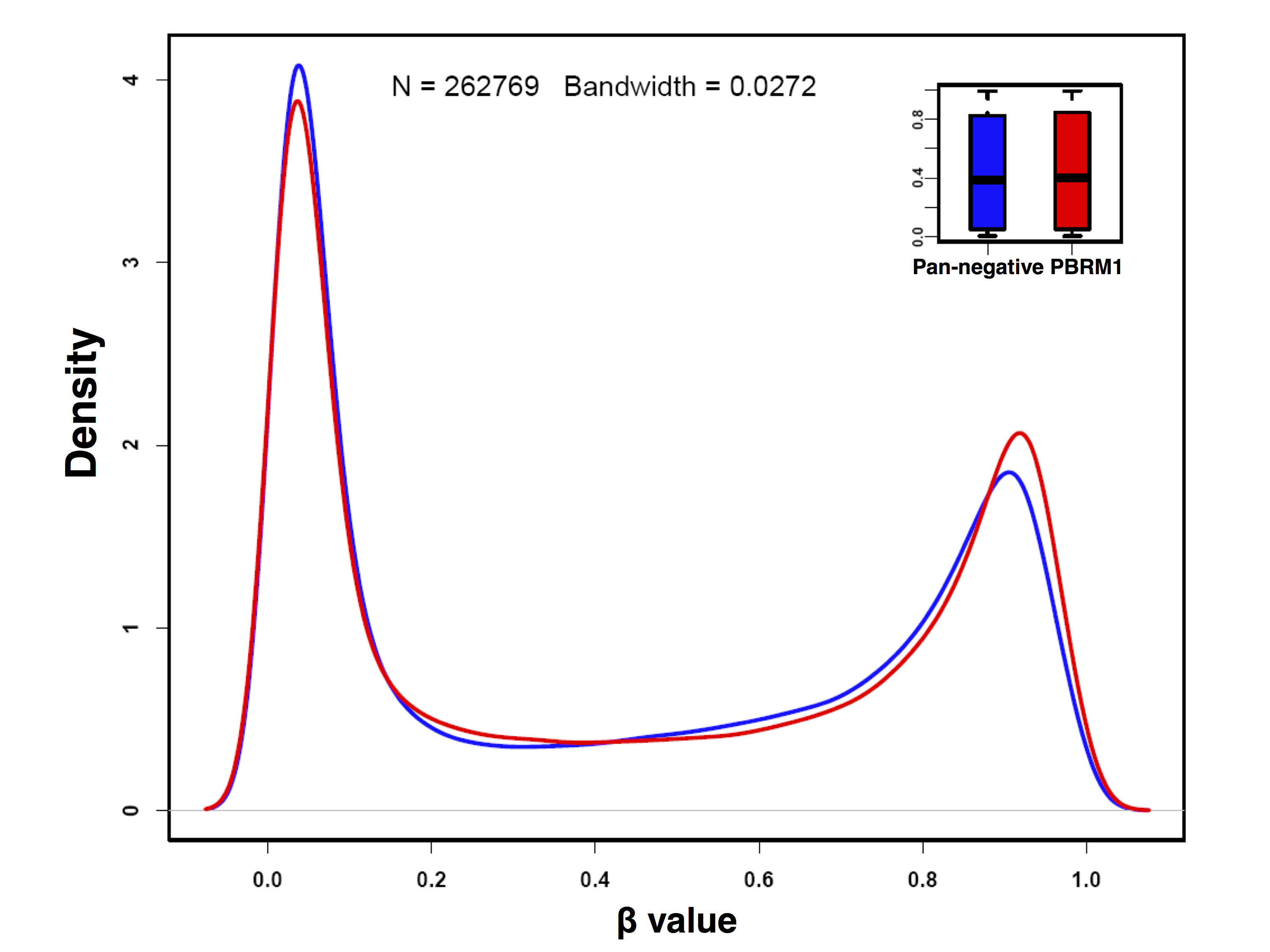
**

**Figure S1 Global methylation density in *PBRM1* mutated group and “pan-negative” group.** Methylation density of *PBRM1* mutated group was shown in red and methylation density of “pan-negative” group was shown in blue.


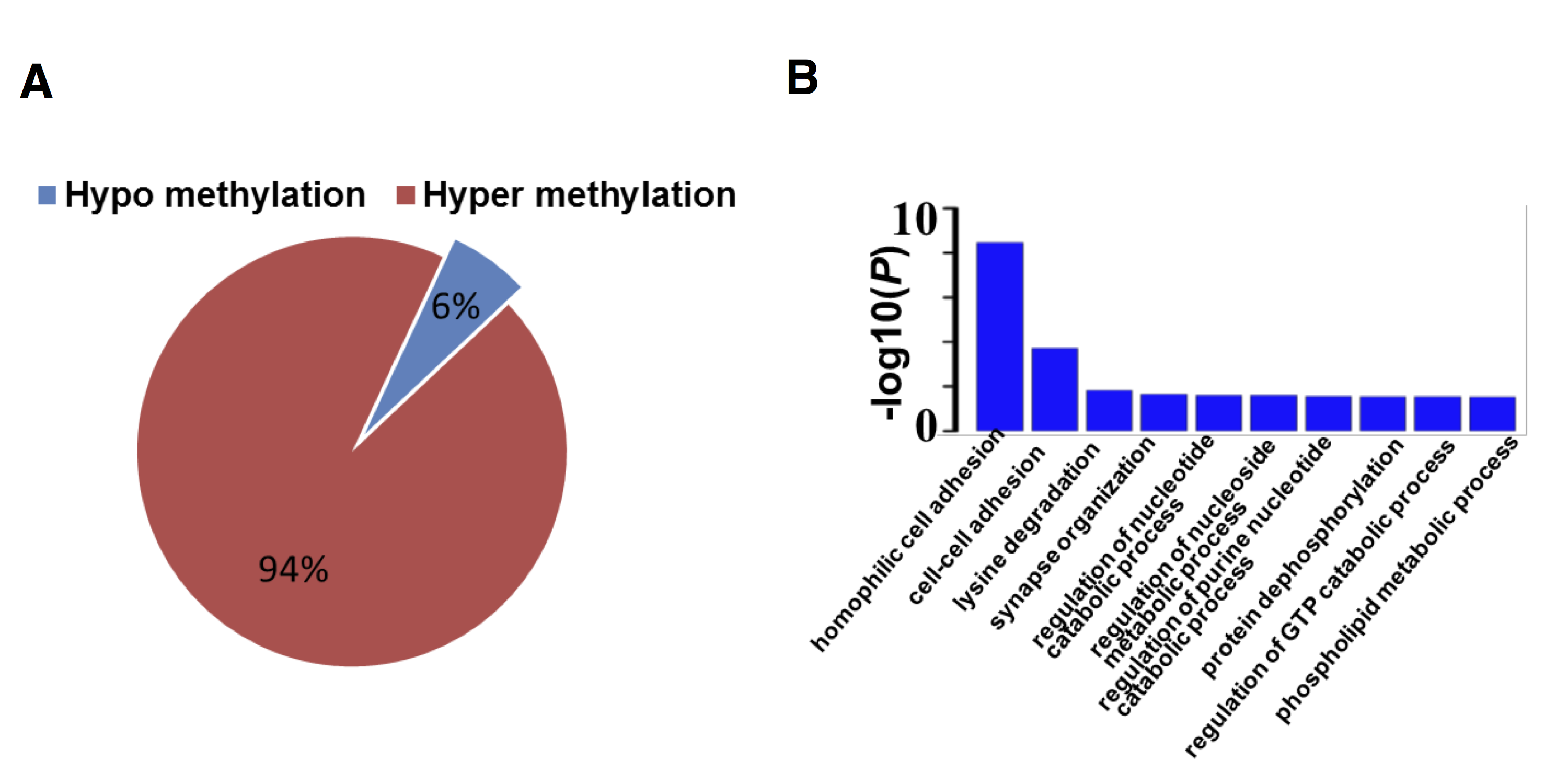


**Figure S2 Statistics results of altered methylated genes numbers and functions.** (A) Pie chart presentation of the percentage of hyper-methylated and hypo-methylated genes in altered methylated genes. (B) Top 10 functional enrichment results of hyper-methylated genes by ClueGO plugin in Cytoscape software.


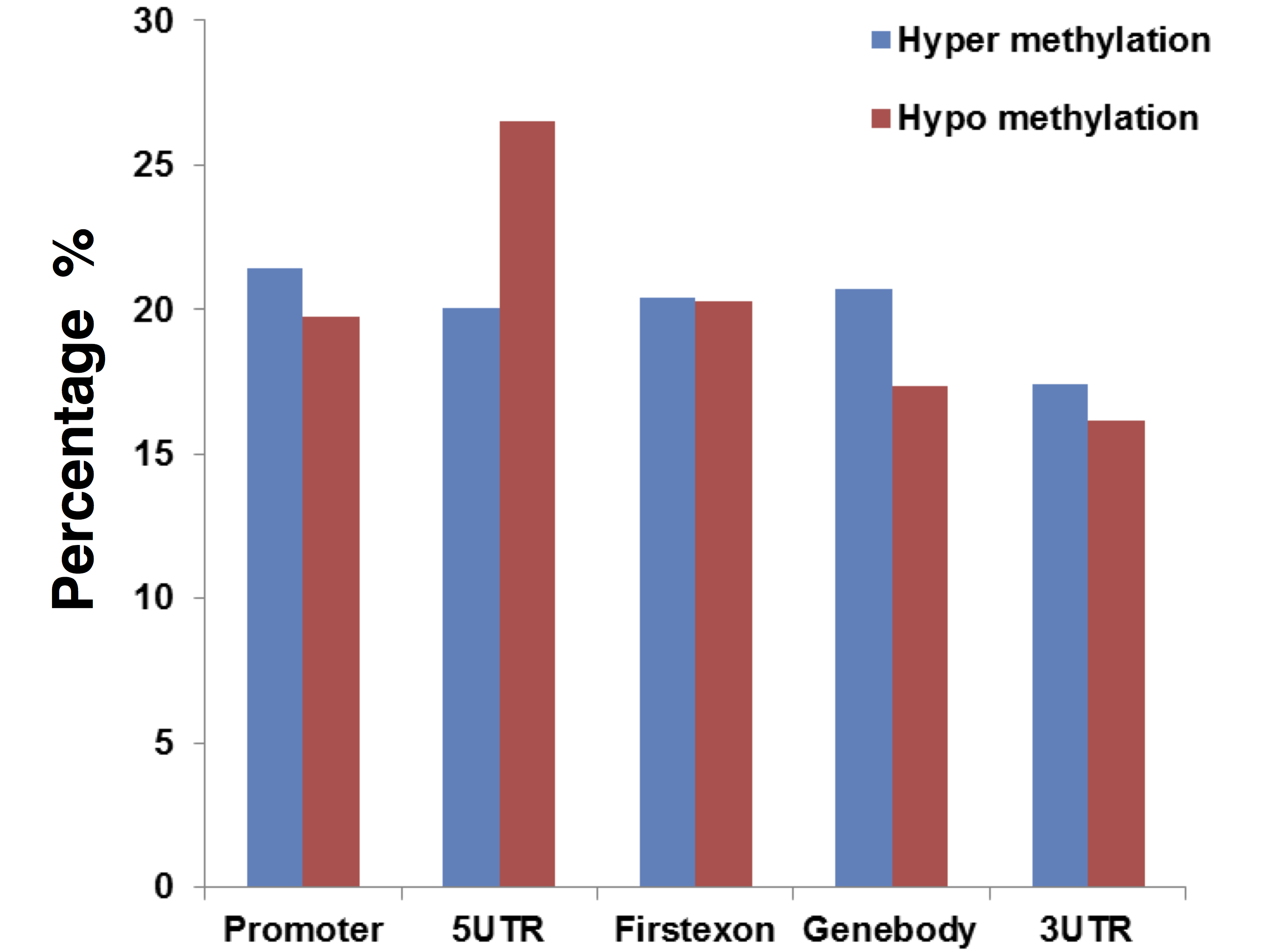


**Figure S3 Percentage of different gene region (promoter, 5`UTR,1^st^ exon, gene body and 3`UTR) in hyper-methylation and hypo-methylation genes.**
